# Genetic Variation within the Mx Gene of Commercially Selected Chicken Lines Reveals Multiple Haplotypes, Recombination and a Protein under Selection Pressure

**DOI:** 10.1371/journal.pone.0108054

**Published:** 2014-09-22

**Authors:** Janet E. Fulton, Jesus Arango, Rizwana A. Ali, Elaine B. Bohorquez, Ashlee R. Lund, Chris M. Ashwell, Petek Settar, Neil P. O'Sullivan, Matthew D. Koci

**Affiliations:** 1 Hy-Line International, Dallas Center, Iowa, United States of America; 2 Prestage Department of Poultry Science, North Carolina State University, Raleigh, North Carolina, United States of America; Instituto de Higiene e Medicina Tropical, Portugal

## Abstract

The Mx protein is one of the best-characterized interferon-stimulated antiviral mediators. Mx homologs have been identified in most vertebrates examined; however, their location within the cell, their level of activity, and the viruses they inhibit vary widely. Recent studies have demonstrated multiple Mx alleles in chickens and some reports have suggested a specific variant (S631N) within exon 14 confers antiviral activity. In the current study, the complete genome of nine elite egg-layer type lines were sequenced and multiple variants of the Mx gene identified. Within the coding region and upstream putative promoter region 36 SNP variants were identified, producing a total of 12 unique haplotypes. Each elite line contained from one to four haplotypes, with many of these haplotypes being found in only one line. Observation of changes in haplotype frequency over generations, as well as recombination, suggested some unknown selection pressure on the Mx gene. Trait association analysis with either individual SNP or haplotypes showed a significant effect of Mx haplotype on several egg production related traits, and on mortality following Marek's disease virus challenge in some lines. Examination of the location of the various SNP within the protein suggests synonymous SNP tend to be found within structural or enzymatic regions of the protein, while non-synonymous SNP are located in less well defined regions. The putative resistance variant N631 was found in five of the 12 haplotypes with an overall frequency of 47% across the nine lines. Two Mx recombinants were identified within the elite populations, indicating that novel variation can arise and be maintained within intensively selected lines. Collectively, these results suggest the conflicting reports in the literature describing the impact of the different SNP on chicken Mx function may be due to the varying context of haplotypes present in the populations studied.

## Introduction

The Myxovirus-resistance (Mx) proteins are interferon-induced, dynamin-like, large GTPases that were first identified because of their association with influenza virus resistance in laboratory mice [1]. Since this initial description, Mx homologues have been described in multiple species. In most animals, at least two Mx genes have been described; however, not all of these different Mx genes have documented antiviral activity (reviewed in [2]). Avian species appear to have just one Mx gene. There is conflicting evidence in the literature on the antiviral properties of the avian Mx proteins. Initial studies of the Mx proteins expressed by chickens and ducks failed to demonstrate antiviral activity [3], [4]. Subsequent studies reported the existence of multiple Mx alleles among different genetic lines of chickens and mouse cell lines transfected with different chicken Mx alleles showed antiviral activity [5]. Fourteen amino acid variants were identified within the Mx protein from multiple chicken breeds, with antiviral activity seemingly linked to one amino acid variant at position 631 (S631N) [5], [6].

Polymorphisms of the Mx gene have been reported in multiple breeds of chickens, including Australorp, Fayoumi, Japanese native chickens, Indonesian native chickens, White Leghorns, Broilers and inbred laboratory lines [5], [7]–[10]. Most of these reports have focused primarily on exon 14 (13^th^ coding exon) and the S631N variant, though other non-synonymous variants have been identified in other exons, and multiple haplotypes have been recognized.

The association between the 631 variant and antiviral activity was investigated in various breeds and with different systems. *In vivo* work by Ewald *et al.* [11] using commercial meat-type (broiler) chicks suggested that those with the N631 variant were more resistant to viral challenge than those with the S631 variant. However, other laboratories using primary chicken embryo fibroblast, transfected cells, chicken embryos, or chicks found no difference in the resistance to influenza virus infection regardless of which 631 variant was expressed [10], [12]–[15]. Furthermore, Schusser *et al.* [14] demonstrated that neither of these Mx variants (N631 or S631) had GTPase activity, which is essential for antiviral activity [16]. Collectively, these results suggest that any antiviral activity expressed by the chicken Mx is likely more complicated than just one amino acid position, especially given the complex structural interactions involved in Mx biology and the numbers of polymorphism reported for the chicken Mx gene.

The significance of variation in the Mx gene and its potential role in resistance to avian influenza is intriguing. We report here variation in the promoter, 5′ untranslated, and coding regions of the Mx gene in 9 elite chicken lines representing the three different breeds used by Hy-Line International for commercial egg production. These chicken lines contribute to over 40% of the commercial egg layer production birds in the world and thus represent a considerable proportion of global commercial egg production.

## Materials and Methods

### Genetic Material

A DNA archive consisting of multiple generations of males (1996–2011) from 9 different chicken lines was utilized for all studies. These lines are the elite pure lines used to produce commercially utilized egg-production chickens. DNA was obtained from blood from 135–1,264 birds from multiple generations of each line. Genotype and phenotype information from 7,964 samples was utilized for all subsequent trait association analyses. These 9 lines encompass three different breeds; six White Leghorn lines (WL) that produce white-shell eggs, one Rhode Island Red derived line (RIR) that produces brown-shell eggs and two White Plymouth Rock derived lines (WPR) that produce brown-shell eggs.

### SNP Identification and Genotyping

Initially twelve individuals from each line were sequenced using Sanger sequencing (BigDye3.1, Applied Biosystems) on an ABI3100 (Applied Biosystems), and the resulting sequence data was analyzed using Vector NTI v. 10 (Invitrogen) to determine the number of potential SNP and haplotypes within these lines. Primer pairs (12) used to produce the template amplicons for sequencing (exons only) are as defined in Table 1. Subsequently, DNA pools (10 individuals per pool, 1 pool per line) were sequenced using the Illumina GAIIx at 7–10x coverage [17]. This resequence data was visualized using IGV viewer, version 2.3.8 [18] which allowed visualization of putative SNP within flanking regions, introns and exons of each line.

**Table 1:** Primers used for amplification and sequencing.

Amplicon	Forward (5′-3′)	Reverse (5′-3′)	Product size (bp)
MxEX_2UTR	AGCACAGTGATCCCTTGGAA	GCACCCCAAAAACTCCTACA	982
MxEX_2	GAGCAAGCCAGAAGAACAGC	TGGAGAGTATCTGTGCCTTTCC	300
MxEX_3	TTTGTTTGTTTCTGGAATCAGC	TCAGGTGAAAACCTGAGAAGG	294
MxEX_4	CCCAGTCCACTCACACAATG	GAGCAAGGGCAATACGCTAC	231
MxEX_5	TGCTTTCCTCTTTCCACCTC	CCTTTGCATAAATTGGCAGA	215
MxEX_6-7	GGAGTGGTCGCATCCTACAT	CAATTCGTTGCAGAAGTCCA	812
MxEX_8	GGCATACTTCCCACAAGCAG	TGAAAGGAAGAAGGGTTGGA	236
MxEX_9-10	CCAGCTGTGTTCAGCCTACC	TCAGCTGCAAGTGATGGTTT	820
MxEX_11	TTCTTTCTTAACCAGAAATTTATGAAG	TGTGTGGCCTGTGAGACGTA	249
MxEX_12	AGATGCCCAGCTATTTCAGC	AGGTTATGGCTTGTCCCTCA	222
MxEX_13	CAGAACTTGTTCTCTTCTTTTCCA	CTGGTGTACTGTGTTGTAGTCTGC	300
MxEX_14	AGCAACTCCATACCGTGTTTT	TGCTAGAAAGCAAAAGCAGAAA	398

The confirmation of SNP was accomplished using fluorescence-based competitive allele specific PCR with KASP chemistry [19]. SNP-specific primer sets were developed using flanking sequence information. Initially genotype information was obtained on individuals from the 1995 or 1996 and 2010 generations. Linkage analysis indicated that specific SNP alleles were found in linkage disequilibrium within the lines. This ultimately defined Mx haplotypes. The minimum number of SNP required to identify haplotypes within each line were then used to genotype the individuals of the remaining generations (1997–2009, and 2011).

### Phenotypic Information

#### Mortality traits

Two mortality related traits were measured. Mortality during grow and lay (LM) were recorded from the sire families which had been placed in multiple field test locations under typical commercial environments. Mortality traits were recorded as sire family means in percent based on 30 daughters per sire, and was measured across generations and genetic lines. The second mortality trait was from multiple generations of a Marek's disease virus (MDV) [20] challenge test also using the progeny testing model. This phenotype was also measured using 30 daughters of each candidate sire. Chicks were maternal antibody positive and were vaccinated at day of age with HVT/SB1 following standard industry practices. At 7 days of age, chicks were inoculated with 500 pfu of the highly virulent strain of serotype 1 MDV (vv+ isolate 686) and mortality due to MDV (MM) was recorded until 17–18 weeks of age [20].

#### Performance Traits

The performance traits recorded are typical for commercial egg production lines and include egg production (egg number, EN and lay rate, PD (%)), sexual maturity (SM, age at onset of lay), and egg quality traits [shell strength (PS, g-force); egg weight (EW, g); albumen height (AH, mm); eggshell color (CO, index using the three parameter L-a-b from the Minolta Chromameter system); and external egg defects (Def, in percent of total eggs produced)]. These traits were also calculated as the mean progeny average for each pure-line sire family across generations and genetic lines [21].

### Statistics

Two sets of statistical models were tested for association between Mx genotype and mortality and performance traits for each genetic line. The first model tested the SNP's allele substitution effects (ASE) for multiple SNP and genetic lines. In this model, the effects of generation (test) and the number of copies of the reference allele for each tested SNP were fit. The second model fit the effects of generation (test) and haplotype for each trait, and was used to test the overall haplotype effect on each trait within line. For those cases with a significant effect, LSM (Least Squares Means) were calculated and separation and test between haplotypes was performed. Analyses were carried out using JMP 11.0 (SAS Institute Inc).

### Analysis of evolutionary selection

Full length nucleotide coding sequence for each of the 12 haplotypes from the commercial lines identified here, plus 53 additional chicken Mx sequences representing multiple breeds reported in GenBank, were analyzed for evidence of recombination as well as site-by-site selection using the Datamonkey webserver (http://www.datamonkey.org) [22]. Briefly, the best fit model (010010) with AIC of 8117.059265150644, also known as the HKY85 model, was determined, followed by analysis to identify individual sites under diversifying or purifying selection using six different methodologies: Mixed Effects Model of Evolution (MEME), Fast Unconstrained Bayesian AppRoximation (FUBAR), Single Likelihood Ancestor Counting (SLAC), Fixed Effects Likelihood (FEL), and Random Effects Likelihood (REL) using Genetic Algorithm for Recombination Detection (GARD) inferred trees. Accession numbers for these additional 53 sequences are: AB088533, AB088534, AB088535, AB088536, AB244818, AY695797, DQ316779, DQ788613, DQ788614, DQ788615, DQ788616, EF575608, EF575609, EF575610, EF575611, EF575612, EF575613, EF575614, EF575615, EF575616, EF575617, EF575618, EF575619, EF575620, EF575621, EF575622, EF575623, EF575624, EF575625, EF575626, EF575627, EF575628, EF575629, EF575630, EF575631, EF575632, EF575633, EF575634, EF575635, EF575636, EF575637, EF575638, EF575639, EF575640, EF575641, EU348752, GQ390353, HM775376, HQ014737, HQ014738, HQ014739, HQ014740, Z23168.

### Structural modeling

The 12 Mx haplotypes identified in the nine elite lines reported here were each analyzed using the RaptorX protein structure prediction server (raptorx.uchicago.edu) which identified protein data bank record 3szr [23] as the most likely structural match. The resulting predicted 3D structures of the 12 chMx haplotypes were visualized using Swiss PDB Viewer 4.1 (http://spdbv.vital-it.ch/).

### Animals

The protocols for all experiments involving the collection of blood samples and phenotypic observations used in this study were reviewed and approved by the Institutional Animal Care and Use Committee (IACUC) at Hy-Line International.

## Results

**Figure 1:**
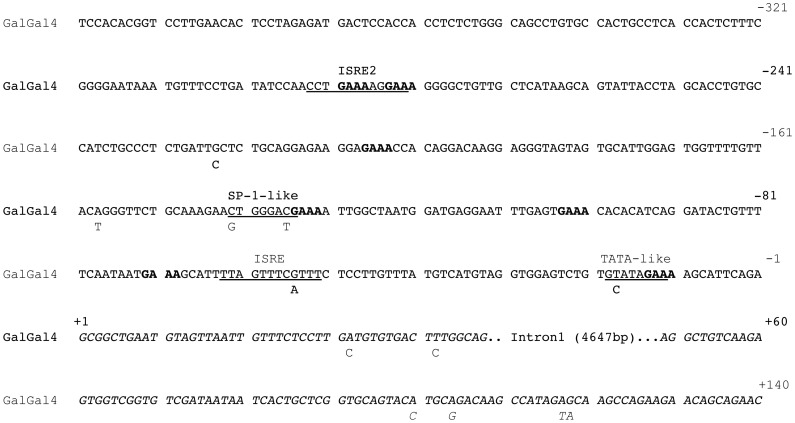
Location of SNP within the promoter and untranslated region of chicken Mx. galGal4 Mx promoter region starting 400 nt upstream of the RNA transcription initiation site through the first 140 nt of the RNA transcript (excluding 4,647 nt from intron 1) are shown above. The previously described ISRE (−52 to −63) as well as other potential functional elements (ISRE2, −282 to −293; SP1-like element, −135 to −142; TATA box-like element, −19 to −12) are underlined. The “GAAA” motif found repeated in many IFN regulated gene promoter regions are shown in bold. SNP found in the 9 elite lines that differ from galGal4 are shown under the reference sequence. Additionally, the RNA transcription initiation and Mx 5′UTR, comprised of exon 1 and the first 92 nt of exon 2, is shown in italics.

Combining the information from the *de novo* sequencing and the NextGen resequence data of the elite commercial lines, revealed multiple SNP within the Mx gene and its immediate upstream region (Table 2). SNP identified within the 400 bp upstream of the start of exon 1 and exonic SNP that changed the amino acid codons were studied in detail. These SNP are listed in Table 2, along with their location within galGal4 from UCSC genome browser (genome.ucsc.edu), their nucleotide change (galGal4 > variant), their codon with amino acid change (if applicable) and the affected predicted protein domain. If the SNP has been previously reported, the source is indicated. There were six SNP identified in the promoter region (4,787 bp upstream of the ATG start in exon 2) and are labeled with the prefix “P”. Among these six SNP, one (MxP-55) lies within the interferon-stimulated response element (ISRE) region previously described by Schumacher *et al.* [24] as essential for Mx gene expression. Of the remaining five, one (MxP-18) falls within a putative TATA-like element, two (MxP-136 and -142) are located within a possible SP-1-like binding site [25], [26], and the other two (MxP-158 and -224) are not associated with any known or proposed functional elements (Figure 1). Within the 140 bp that make up the 5′ untranslated region, six SNP were identified, two in exon 1 and four in exon 2. These are labeled with the prefix of “Mx5U” (for 5′ untranslated) with the numeric label indicating how many bases they are from the beginning of the RNA transcription initiation (Table 2 and Figure 1). Within the actual coding region of the Mx gene, 24 SNP were found within the nine elite lines (Table 2). These SNP were then confirmed by SNP-PCR. There were four additional SNP (indicated by * Table 2) that were reported in the literature [3], [5], [8], [13] but not found to be segregating within the populations in this study. The coding-region SNP were named based on the affected nucleotide position relative to the ATG start codon of Mx. Multiple SNP were also found within the introns of Mx. These were not genotyped except when necessary to identify the regions of recombination. In total, three novel SNP were identified in the nine elite lines, one in the 5′ UTR, and two non-synonymous substitutions (dNS) SNP located in the distal stalk region (Table 2).

**Table 2:** SNP genotyped, their location within the gene, position within galGal version 4, nucleotide change, and codon affected, and the MX protein domain involved.

SNP	Location	GGA1 bp	nt change^1^	Codon	AA	Domain^3^	Ref
MxP-224^2^	Promoter	108,275,357	G>C	-	-	-	[24], [36]
MxP-158	Promoter	108,275,433	A>T	-	-	-	[36]
MxP-142	Promoter	108,275,449	C>G	-	-	-	[36]
MxP-136	Promoter	108,275,455	C>T	-	-	-	[36]
MxP-55	Promoter	108,275,536	G>A	-	-	-	[36]
MxP-18	Promoter	108,275,573	T>C	-	-	-	[36]
Mx5U32^2^	Exon 1	108,275,623	A>C	-	-	-	[24], [36]
Mx5U42^2^	Exon 1	108,275,633	T>C	-	-	-	[24], [36]
Mx5U100	Exon 2	108,280,332	A>C	-	-	-	[24]
Mx5U104	Exon 2	108,280,336	A>G	-	-	-	[24], [36]
Mx5U117	Exon 2	108,280,349	A>T	-	-	-	
Mx5U118	Exon 2	108,280,350	G>A	-	-	-	[24]
MxCDS13	Exon 2	108,280,385	C>T	CGG>TGG	R5W	ND	[5], [13]
MxCDS62	Exon 2	108,280,434	G>A	CGG>CAG	R21Q	ND	[5], [8], [10], [13]
MxCDS122	Exon 2	108,280,494	C>G	CCA>CGA	P41R	ND	[5], [8], [10], [13]
MxCDS125	Exon 2	108,280,497	T>C	TTA>TCA	L42S	ND	[5], [8], [10], [13]
MxCDS156	Exon 2	108,280,528	T>A	GCT>GCA	A52A	ND	[5], [8], [10]
MxCDS254*	Exon 3	108,282,234	T>C	TTG>TCG	L85S	ND	[5]
MxCDS281	Exon 3	108,282,261	G>A	CGA>CAA	R94Q	BSE1	[5], [10]
MxCDS351	Exon 3	108,282,331	T>C	ATT>ATC	I117I	G-domain	[5], [8]
MxCDS554*	Exon 4	108,283,451	A>G	AAA>AGA	K185R	G-domain	[5], [13]
MxCDS595*	Exon 5	108,285,185	G>A	GGT>AGT	G199S	G-domain	[5], [13]
MxCDS605	Exon 5	108,285,194	G>C	AGT>ACT	S202T	G-domain	[5], [8], [13]
MxCDS694	Exon 5	108,285,283	G>A	GGG>AGG	G232R	G-domain	[5]
MxCDS696	Exon 5	108,285,285	G>C	GGG>GGC	G232G	G-domain	[5], [8], [13]
MxCDS792	Exon 6	108,286,581	T>C	AAT>AAC	N264N	G-domain	[5], [10]
MxCDS813	Exon 6	108,286,602	A>G	GAA>GAG	E271E	G-domain	[5], [10], [13]
MxCDS922	Exon 7	108,287,175	G>A	GTA>ATA	V308I	G-domain	[5], [8], [10], [13]
MxCDS1015	Exon 7	108,287,268	A>G	ACA>GCA	T339A	G-domain	[5], [8], [10], [13]
MxCDS1203*	Exon 9	108,289,299	A>C>G	CTA>CTC>CTG	L401L	BSE2	[3], [5], [8]
MxCDS1248	Exon 9	108,289,344	A>G	ACA>ACG	T416T	MD	[5], [8]
MxCDS1329	Exon 10	108,289,898	C>T	AAC>AAT	N443N	MD	[5]
MxCDS1455	Exon 11	108,290,956	C>T	CGC>CGT	R485R	MD	[5], [8]
MxCDS1545	Exon 11	108,291,046	G>A	GCG>GCA	A515A	MD	[5], [8], [10]
MxCDS1643	Exon 13	108,293,825	C>T	GCC>GTC	A548V	MD	[5], [8], [13]
MxCDS1747	Exon 13	108,293,929	A>G	ACA>GCA	T583A	MD/GED	[5], [10], [13]
MxCDS1801	Exon 13	108,293,983	A>C	AAA>CAA	K601Q	MD/GED	
MxCDS1892	Exon 14	108,295,901	G>A	AGT>AAT	S631N	GED	[5], [8], [13]
MxCDS1905	Exon 14	108,295,914	C>A	AGC>AGA	S635R	GED	
MxCDS2019	Exon 14	108,296,028	G>A	CAG>CAA	Q673Q	Hinge1	[5], [8]

1 galGal4 > variant.

2 Denotes SNP that were reported in the literature, but were not genotyped in the elite lines. Examination of the resequence data indicates that all haplotypes had the variant allele at these SNP.

3 Mx protein domain; ND  =  domain not determined, BSE  =  bundle signaling element, G-domain  =  GTPase domain, MD  =  Middle domain, GED  =  GTPase effector domain, MD/GED  =  transition region between MD and GED, Hinge  =  protine hinge region.

*Denotes SNP previously reported in the literature and found not segregating in the elite lines.

### Haplotypes

Each line was genotyped for 33 SNP on 135 to 200 animals per generation from widely separated generations. This resulted in the identification of 12 Mx haplotypes across all the lines (Table 3), eight of which, to the best of our knowledge, have not been previously reported. The haplotype for the reference genome (gagGal4 UCSC) is provided for comparison.

**Table 3:** Mx haplotypes identified in the 9 elite lines as determined by 6 promoter, and 34 exon SNP.

Haplotype	MxP-224^1^	MxP-158	MxP-142	MxP-136	MxP-55	MxP-18	Mx5U32^1^	Mx5U42^1^	Mx5U100	Mx5U104	Mx5U117	Mx5U118	MxCDS13	MxCDS62	MxCDS122	MxCDS125	MxCDS156	MxCDS254*	MxCDS281	MxCDS351	MxCDS554*	MxCDS595*	MxCDS605	MxCDS694	MxCDS696	MxCDS792	MxCDS813	MxCDS922	MxCDS1015	MxCDS1203*	MxCDS1248	MxCDS1329	MxCDS1455	MxCDS1545	MxCDS1643	MxCDS1747	MxCDS1801	MxCDS1892	MxCDS1905	MxCDS2019
galGal4	G	A	C	C	G	T	A	T	A	A	A	G	C	G	C	T	T	T	G	T	A	G	G	G	G	T	A	G	A	A	A	C	C	G	C	A	A	G	C	G
Mx-H01	C	A	C	C	G	T	C	C	A	G	T	G	C	A	C	T	A	T	G	C	A	G	G	G	G	T	A	A	G	A	A	C	T	A	C	A	A	G	C	G
Mx-H02	C	A	C	C	G	T	C	C	A	G	T	G	C	A	C	T	A	T	G	C	A	G	G	G	G	T	A	G	A	A	A	C	T	A	T	A	A	A	C	G
Mx-H03	C	A	C	T	G	T	C	C	A	G	A	G	C	A	C	T	T	T	G	T	A	G	C	G	G	T	A	A	G	A	A	T	C	G	C	A	A	G	C	G
Mx-H04	C	T	G	C	A	C	C	C	A	G	A	G	C	A	G	C	A	T	G	C	A	G	G	G	G	T	A	G	A	A	G	C	T	A	C	A	A	G	C	G
Mx-H05	C	A	C	C	G	T	C	C	A	G	A	G	C	A	G	C	A	T	G	T	A	G	G	G	G	T	A	G	A	A	A	C	T	A	T	A	A	A	C	G
Mx-H06	C	A	C	C	G	T	C	C	A	A	A	G	C	G	C	T	T	T	G	C	A	G	G	G	G	T	A	G	A	A	G	C	T	A	T	A	A	A	C	A
Mx-H07	C	A	C	C	G	T	C	C	A	A	A	G	C	G	C	T	T	T	G	C	A	G	G	G	G	T	A	G	A	A	G	T	C	G	C	A	A	G	C	G
Mx-H08	C	A	C	C	G	T	C	C	A	A	A	G	C	G	C	T	T	T	G	T	A	G	G	A	G	T	A	G	A	A	G	C	T	A	T	A	A	A	C	A
Mx-H09	C	A	C	C	G	T	C	C	A	A	A	G	C	G	C	T	T	T	G	T	A	G	G	G	G	T	A	G	A	A	A	C	C	G	C	A	C	G	C	G
Mx-H10	C	A	C	C	G	T	C	C	A	A	A	G	C	G	C	T	T	T	G	T	A	G	G	G	C	T	A	G	A	A	G	C	C	G	C	A	A	A	C	G
Mx-H11	C	A	C	T	G	C	C	C	C	G	A	A	T	A	G	C	A	T	A	T	A	G	G	G	G	C	G	A	G	A	G	C	C	G	C	G	A	G	A	G
Mx-H12	C	A	C	C	G	T	C	C	A	A	A	G	C	G	C	T	T	T	G	T	A	G	G	A	G	T	A	G	A	A	G	C	T	A	C	A	A	G	C	G

1 Denotes SNP that were not tested within the elite lines, not segregating in the elite lines.

MxP-# denotes SNP in the promoter regions 400 bp upstream of RNA transcription initiation which includes the Mx ISRE. Numbers indicates nt position upstream of RNA initiation.

Mx5U# denotes SNP in the 5′ untranslated region. Number indicates nt position within the mRNA transcript.

MxCDS# denotes SNP in the coding region of Mx. Number indicates nt position from the beginning of the start codon.

*Denotes SNP not found within the 9 elite lines examined in this study but have been previously been reported.

### Mx Variation

Each elite line contained from one to four haplotypes (Table 4). Within the elite stocks tested here, many of the haplotypes appear to be breed specific, with the exception of haplotype Mx-H04, which was found in both the RIR and WPR breeds. Investigation of the historical haplotype segregation within the nine elite lines from 1995 to 2010 indicated that five of the eight lines that were segregating for Mx haplotypes had significant changes in Mx haplotype frequency during this time.

**Table 4:** Mx haplotype frequency changes over time by line.

		Frequency		
Line	Haplotype	1995/1996	2010	P value
WL-01	Mx-H01	0.82	0.80	ns^1^
	Mx-H08	0.17	0.19	ns
	Mx-H12	0.01	0.01	ns
WL-02	Mx-H05	0.41	0.64	<0.0001
	Mx-H11	0.21	0.36	<0.0001
	Mx-H01	0.38	0.00	<0.0001
WL-03	Mx-H06	0.54	0.84	<0.0001
	Mx-H08	0.40	0.14	<0.0001
	Mx-H05	0.06	0.02	ns
WL-04	Mx-H05	0.46	0.78	<0.0001
	Mx-H02	0.00	0.04	ns
	Mx-H01	0.53	0.18	<0.0001
	Mx-H06	0.01	0.00	ns
WL-05	Mx-H06	1.00	1.00	ns
WL-06	Mx-H01	0.86	0.98	<0.0001
	Mx-H05	0.14	0.02	<0.0001
WPR-01	Mx-H10	0.48	0.47	ns
	Mx-H04	0.23	0.28	ns
	Mx-H09	0.30	0.24	ns
WPR-02	Mx-H10	0.84	0.96	0.0032
	Mx-H09	0.10	0.02	0.0006
	Mx-H04	0.06	0.02	ns
RIR-01	Mx-H03	0.67	0.62	ns
	Mx-H04	0.30	0.36	ns
	Mx-H07	0.03	0.02	ns

1 not significant.

### Mx Recombinants

Close examination of the SNP composition of each haplotype revealed that two haplotypes appeared to be the result of within-line recombination events (Table 3). Haplotype Mx-H02 was found in low frequency in only one line (WL-04). It is a recombination of the two major haplotypes within line WL-04, having the same SNP composition for the promoter region through SNP MxCDS351 (exon 4) as MH-H01 and the same SNP composition from SNP MxCDS992 (exon 8) to the end of the gene as MX-H05. The seven intervening exonic SNP are identical between the two parental haplotypes. Further genotyping with the numerous intronic SNP that differ between these two haplotypes allowed the actual recombination region to be narrowed down to a 450 bp region within intron 5 (data not shown). The *de novo* occurrence of this haplotype was tracked to a female from the 2003 generation. It has gradually increased in frequency since that time, reaching 0.04 by 2010. The low numbers of individuals with this haplotype are insufficient to determine if there are any trait associations, though the continued increase in frequency is suggestive of a selective advantage.

A second recombinant was identified in line WL-01 and appears to be the result of recombination between the two major haplotypes within that line (Table 3). Haplotype Mx-H12 is identical to Mx-H08 from the promoter through SNP MxCDS1248 (exon 10) and identical to haplotype Mx-H01 from SNP MxCDS1643 (exon 14) through to the end of the gene. The intervening exonic SNP are identical between the two parental haplotypes. The use of intronic SNP that differ between the two parental haplotypes narrowed the identification of the actual recombinant region to a 414 b region of intron 11. Haplotype Mx-H12 has been maintained at a low frequency (<0.04) since the archive DNA collection was initiated in 1996, thus the original progenitor could not be identified.

### Trait Associations

#### Trait Associations with SNP (ASE)

Those Mx SNP that had a significant association with phenotype are summarized in Table 5, along with the size of the effect and which SNP allele was favorable. Allele substitution effects were found in 4 lines. For two mortality traits, allele specific effects were found with five SNP (MxP-55, MxCDS62, MxCDS122, MxCDS694, and MxCDS1015) in four lines (WL-02, WL-03, WPR-01 and RIR-01) The most consistent association was for MxCDS122, which showed significance in three lines, and in one line (WL-03) the ASE was significant for progeny mortality in both the MDV challenge test and during the grow/lay period.

**Table 5:** Mx SNP with a significant allele substitution effect, and allele with favorable effect by trait.

Trait	Line	SNP	Estimate	SE	P>|t|	Favorable Allele
Mortality^1^						
MM	WL-02	MxCDS1015	−1.51	0.697	0.031	G
	WL-03	MxCDS122	−1.52	0.715	0.033	G
	WL-03	MxCDS694	1.36	0.684	0.047	G
	WPR-01	MxP-55	1.52	0.715	0.033	A
LM	WL-02	MxCDS122	−1.14	0.037	0.002	G
	WL-03	MxCDS122	1.59	0.795	0.0457	C
	RIR-01	MxCDS62	3.25	1.471	0.0219	A
Performance^2^						
EN	WL-03	MxCDS122	−1.14	0.527	0.031	C
PD	WL-03	MxCDS122	−0.93	0.326	0.045	C
	WL-03	MxCDS351	−0.25	0.093	0.006	C
	WL-03	MxCDS694	0.19	0.096	0.047	G
	RIR-01	MxCDS62	0.58	0.215	0.007	G
AH	WL-02	MxCDS122	0.07	0.032	0.031	G
	WL-02	MxCDS1248	0.07	0.002	0.002	G
CO	WL-02	MxCDS122	−0.16	0.039	<0.0001	G
	WL-02	MxCDS1015	0.14	0.023	<0.0001	A
	WL-02	MxCDS1248	0.11	0.026	<0.0001	A
	WL-03	MxCDS351	0.13	0.024	<0.0001	C
	WL-03	MxCDS694	−0.13	0.025	<0.0001	G
EW	WL-03	MxCDS351	0.37	0.071	<0.0001	T
	WL-03	MxCDS694	−0.39	0.073	<0.0001	A
PS	WL-02	MxCDS1248	1.65	0.815	0.044	G
Def	RIR-01	MxCDS62	−0.32	0.123	0.010	A

1 MM: mortality (%) due to MDV challenge; LM: mortality (%) during lay in commercial environments.

2 EN: number of eggs produced, PD: rate of lay (%), AH: albumen height (mm), CO: shell color index, EW: egg weight (g), PS: shell resistance (N), Def: external egg defects.

The ASE on performance traits also identified several SNP with significant effects in three lines. Most of the significant associations were for egg production (either egg number or lay rate) and egg shell color (seen in two lines). Significant ASE for egg weight and albumen height was seen in two lines, whereas shell puncture resistance and egg defects each had only one instance of significant association.

#### Trait Associations with Haplotypes

Significant haplotype effects found in each line are summarized by trait in Table 6. The favorable haplotype is indicated first, and the size of the effect for the favorable vs alternate haplotype is given. Significant associations with mortality (MDV challenge and during the grow/lay period) were found in one line (WL-02). The size of the effect for mortality due to Marek's Disease Virus was a decrease in progeny mortality of 5.21% for haplotype H11 vs H05 that was found in the sire and a decrease in progeny mortality of 1.8% during the grow/lay period in commercial environments.

**Table 6:** Mx haplotypes with significant effect.

Trait	Line	Prob.>F	Extreme Difference^1^	Size of effect
Mortality^2^				
MM	WL-02	0.027	H1111-H0505	−5.21
LM	WL-02	0.018	H1111-H0505	−1.8
Performance^3^				
SM	WPR-01	0.050	H0409-H0404	0.77
PD	WL-03	0.022	H0606-H0506	0.92
AH	WL-02	0.038	H1111-H0105	0.17
CO	WL-02	<0.0001	H1111-H0505	0.32
	WL-03	<0.0001	H0808-H0606	0.26
EW	WL-03	<0.0001	H0608-H0606	0.49
PS	WL-04	0.039	H0506-H0106	21.7

1 First haplotype has the most favorable effect for each trait.

2 MM: mortality (%) due to MDV challenge; LM: mortality (%) during lay in commercial environments.

3 SM: sexual maturity (age at first egg), PD: production rate (%), AH: albumen height (mm), CO: shell color index, EW: egg weight (g), PS: shell resistance (N).

Trait association between haplotypes and performance traits of progeny showed at least one significant association (p<.05) in four of the lines. Haplotype association with shell color was found in two lines and was highly significant (p<.0001) in both lines. Haplotype 11 showed consistent advantage for four traits (two mortality and two performance traits).

### Evidence of Selection

Full length coding sequence of the 12 Mx haplotypes identified herein, were aligned with 53 additional chicken Mx sequences obtained from GenBank. Overall, these sequences resulted in the identification of 72 SNP within the Mx cds (data not shown). These sequences were then analyzed for individual codons with evidence of either purifying (synonymous substitutions (dS) > dNS) or diversifying (dN > dS) selection using various models. Twenty codons were identified by at least one of the six models, 11 with evidence for diversifying selection and nine with evidence of purifying selection (Table 7). Of these 20, 16 codons correspond with SNP identified in the nine elite lines, and encompass five of the seven SNP associated with performance traits (Table 5). Of the two remaining SNP, one (MxP-55) is located in the promoter and therefore was not analyzed for codon selection, and the second (MxCDS694) corresponds with codon 232, which has evidence of being under purifying selection via MxCDS696.

**Table 7:** Evidence of sites within the chicken Mx under selection.

	Codons Under Selection^a^																		
	Diversifying Selection																		
Model	8	21		41		42		202		308		339		505		539		548	631
SLAC^b^	ns^e^	ns		ns		ns		Ns		ns		ns		ns		ns		ns	ns
FEL^b^	ns	ns		0.079		ns		Ns		ns		ns		ns		ns		ns	ns
IFEL^b^	ns	ns		0.071		ns		ns		ns		ns		0.047		ns		ns	ns
REL^c^	528	325		20,769		661		648		4896		68		1074		813		10,810	706
FUBAR^d^	ns	ns		0.963		0.902		ns		0.953		ns		0.983		0.909		0.968	ns
MEME^b^	ns	ns		0.018		ns		0.077		ns		ns		ns		0.054		ns	ns
	Purifying Selection																		
	117		232		373		401		416		443		485		515		673		
SLAC^b^	<0.001		ns		ns		Ns		0.001		0.019		0.004		0.037		ns		
FEL^b^	<0.001		ns		0.099		Ns		<0.001		0.007		0.001		0.006		0.076		
IFEL^b^	<0.001		0.098		ns		Ns		0.004		0.043		0.011		0.041		ns		
REL^c^	3.8×10^24^		ns		ns		Ns		4.5×10^9^		3822		3.6×10^7^		2926		ns		
FUBAR^d^	0.997		ns		ns		0.949*		1.000		0.998		1.000		0.998		0.972		

a chicken Mx codons with evidence of diversifying selection (dNS > dS) or purifying selection (dS > dNS).

b p-value, *significance ≤0.1.

c Bayes factor, * significance >50 significant.

d Posterior probability, *significance ≥0.9.

e not significant.

### SNP Association With Mx Structural Elements

**Figure 2:**
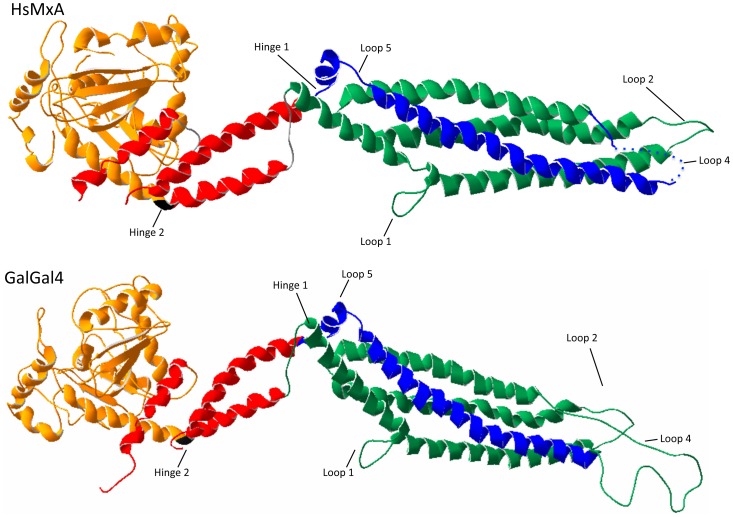
Predicted chicken Mx structure. The chicken Mx sequences were each analyzed using the RaptorX protein structure prediction server. These results identified the crystal structure of human MxA (PDB ID: 3SZR) as the most closely related to the chicken Mx sequence. These results where then visualized using PolyMol, and the regions of the HsMxA (aa#s) and chicken Mx (aa#s) compared. As before the GTPase Domain is shown in orange, the bundle signaling elements are in red, the stalk region which is comprised of the central dyanmin region and the GTPase effector domain are shown in green and blue respectively. The conserved proline residue that forms the hinge between the G-domain and the second BSE is shown in black.

**Figure 3:**
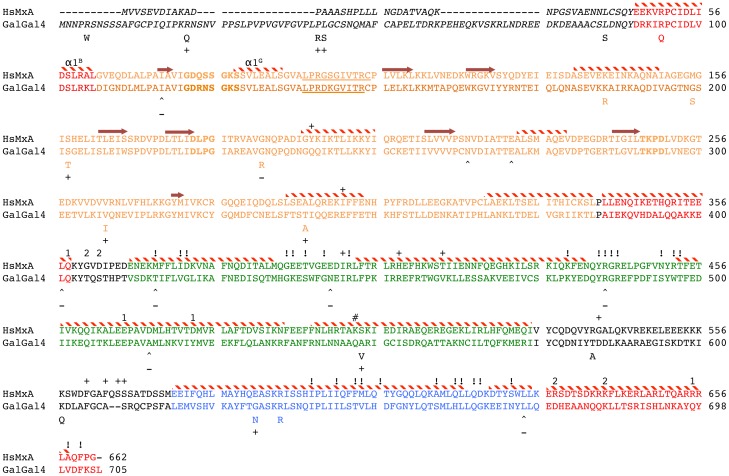
Amino acid alignment of the HsMxA and galGal4. The different Mx functional domains are represented in colored text (BSE  =  red, G-domain  =  orange, Middle stalk domain  =  green, GTPase effector domain  =  blue) with the N-terminal region not represented in shown in italics, and the loop regions that connect BSE2 to MD (L1^BS^) and MD to GED (L4^S^) in plain text. Positions in orange bold represent the conserved GTPase enzymatic domain and underlined orange text denotes the GTP binding site. Secondary structural elements as described by Gao et al. [23] et al. are indicated above the HsMxA sequence. Alpha helices are represented by red and with stripped bars. Beta strands are represented by arrows. Amino acids with similar numbers above the alignment indicate positions described to interact during oligimerization. HsMxA positions labeled with “#” indicates amino acid with forms hydrogen bonds with the backbone of the α3^B^, and “!” denote additional positions described to be involved in oligemerization. “+” above the hsMxA indicates amino acid described by Mitchell et al. [30] as under diversifying selection among primate MxA sequences. Amino acid positions in the chMx associated with dNS changes are reflected by the alternate amino acid under the chMx sequence. Positions associated with dS nt changes are indicated by “∧”. chMx positions with evidence of diversifying (dNS) or purifying (dS) selection are indicated with “+” or “−” under the chMx sequence.

The RaptorX protein structure prediction server [27] was used to infer the location of SNP within potential structural regions. The crystal structure of the human MxA protein reported by Gao *et al.* [23] was identified as the best fit. Based on this tertiary structure, a similar 3D structure was predicted for the chicken Mx protein (Figure 2). It should be noted that the structure reported by Gao *et al.* [23] starts at Tyr45 within the hsMxA sequence and has four regions within the G-domain and one in the loop L4^s^ that were not resolved. Also, as the chicken Mx has approximately 40 additional N-terminal amino acids not found in the mammalian Mx proteins [28], the structure predicted by the RaptorX server starts with Ser84. The chicken Mx protein is predicted to have a similar number of alpha helices, beta sheets, and loop regions (Figure 2 and 3). Examination of the location of these dS and dNS SNP within the Mx protein structure demonstrate that these changes tend to be distributed across the whole sequence, but with a tendency for the dNS SNP to be located at the N-terminal end and the dS SNP more concentrated in the middle domain (MD) (Figure 3).

**Figure 4:**
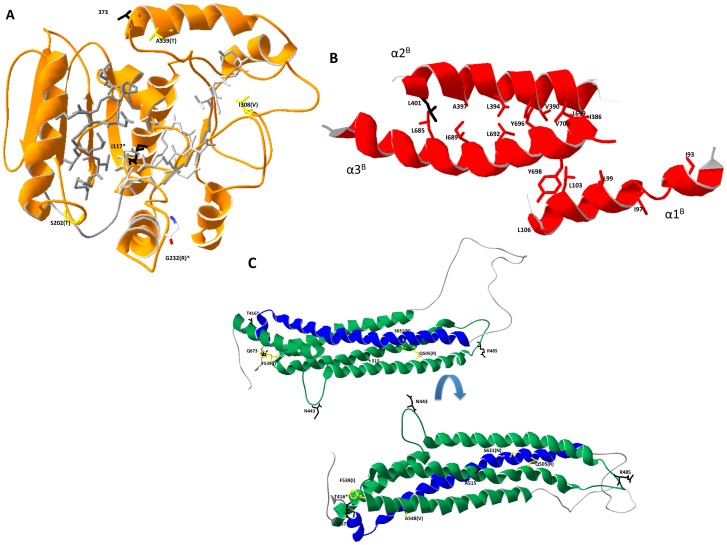
Ribbon structure of the three main functional domains of the Mx protein. (A) Ribbon structure of the predicted chMx G-domain. The amino acids associated with the GTPase active site are shown in light gray and those associated with GTP binding in dark gray. The amino acids found to be under selection and/or associated with traits are depicted in black (dS) or yellow (dNS). Amino acid 232 had 2 SNP associated with it, one dS and one dNS and is shown in red white and blue. The amino acid and position numbers are next to each selected site with the alternate amino acid indicated in parentheses if applicable. Positions that were associated with both traits and selection are denoted with “*”. (B) Ribbon structure of the interacting BSE elements based on the predicted structure. Position 401, with evidence of purifying selection, is shown in black along with the conserved hydrophobic residues associated with the Lucien zipper (red) interactions. (C) Ribbon structure of the predicted stalk domain. The amino acids found to be under selection and/or associated with traits are depicted in black (dS) or yellow (dNS). The structure was rotated 180° (top vs bottom) in order to be able to visualize all the affected position in the stalk.

Examination of these different SNP within the context of functional domains demonstrated several dS and dNS SNP surrounding the GTPase active site within the G-domain (ueure 3 and 4A). While this structure was not fully resolved for MxA [23], six SNP that were found to be associated with performance traits or under selection appear to be located within the G-domain and are clustered around the GTPase active site (Table 5 and Figure 4A). Of these six sites, two were associated with both performance traits and selection (codon 117 and 232). Interestingly codon 232 contains two SNP. SNP MxCDS694 is associated with a dNS change and was found to be associated with performance traits, whereas SNP MxCDS696 does not result in an amino acid change and was associated with purifying selection (Table 5 and 7).

The end of the G-domain is defined by the conserved P384 that forms hinge 2 (Figure 2) and marks the start of the second of three bundle signaling element (BSE) regions [23]. The three BSEs contain a high number of hydrophobic residues, encompassing between 35–42% of amino acids in this region and appear to interact (Figure 4B). As was described for hsMxA, there appeared to be more interactions between α2^B^ and α3^B^ than with α1^B^, with L401, which was found to be under purifying selection (Table 7), interacting with the leucine residues of the leucine zipper in α3^B^ (Figure 4B) [23].

After the central BSE region, the protein forms a large stalk region comprised of the middle domain (MD) and the GTP effector domain (GED) (Figure 4C). From the loop region (L1^BS^) that forms the transition between BSE2 and stalk there are 4 α-helices and 3 loop regions (α1N^S^, L1^S^, α1C^S^, L2^S^, α2^S^, L3^S^, and α3^S^) that make up the MD portion of the stalk. This is followed by loop L4^S^ and serves as the transition between the MD and GED regions of the stalk. The GED then contains an additional 2 α-helices and 1 loop (α4^S^, L5^S^, and α5^S^,) before a final loop that connects the stalk with BSE3 (L2^BS^) (Figure 4C). This region plays key roles in Mx oligomerization [23], [29] and virus specificity [30]. Comparing the amino acid sequences of chicken and hsMxA (Figure 3), one observes that many of the residues reported to be important for oligomerization are clustered in α1N^S^, L1^S^, and L2^S^ of the MD as well as the C-terminal end of α4^S^, L5^S^, and α5^S^ of the GED. These regions correspond with the majority of chMx codons identified as being under purifying selection (Figure 4 and Table 7).

Alternatively, four codons in the stalk region were identified as being under diversifying selection (Table 7). Three of these dNS sites are located in the α2^S^, L3^S^, α3^S^ region of the MD, with the one remaining site (S631N) located in α4^S^ of the GED (Figure 3 and 4C). Only two (A548V and S631N) of these four sites were found to differ among the nine elite lines examined as part of this study (Table 7). The other two sites were identified based on sequence alignments including all full-length chMx sequences.

## Discussion

The Mx genes, and the large GTPase protein they encode, are among the best-studied interferon-stimulated antiviral effector molecules. Their identity, and even their name, is based on their ability to inhibit virus replication, specifically influenza virus. Roughly 10 years after the Mx genes of chickens and ducks were first identified and reported to have no antiviral activity [3], [4], studies by Ko *et al.* [5] examined 15 different breeds and identified 25 SNP resulting in 19 different haplotypes. These 19 haplotypes were then cloned and expressed in mammalian cell lines to assess their antiviral activity. The results of these analyses suggested that some chicken Mx alleles may have antiviral activity and this putative activity appeared to be conferred by the SNP MxCDS1892 (note: Ko *et al.* reported this SNP as 2032 as they numbered from the start of the mRNA), resulting in a change from serine to asparagine at amino acid 631 [5].

Since this initial observation, several laboratories have surveyed various poultry populations and reported over 72 potential SNP either in the literature or in the GenBank database (data not shown). However, most efforts have focused on MxCDS1892, which is often referred to as the “resistance allele” [9]. Surveys of various native, commercial, and laboratory strains of chickens have reported rates of the “resistant allele” ranging from 59.2% to 72.4% and have suggested that the native breeds have a higher frequency of the “resistant allele” than commercial production birds [9], [31], [32]. Limited information, if any, was presented on haplotype information within these breeds.

This current study surveyed and calculated the frequency for SNP found within nine elite commercial egg production lines and analyzed them for association with various mortality and performance traits. Out of the 36 SNP identified in these genetic lines, seven SNP were significantly associated with one or more traits; however, interestingly MxCDS1892 was not among those seven. Examination of each of these seven SNP and the favorable allele for each trait indicates that, within a given line, the same SNP may be associated with multiple traits but have different favorable alleles for each; making it difficult, if not impossible, to understand its true biological significance.

There are multiple reports of Mx variation in different chicken lines. These previous studies attempted to correlate “functional” variants to observations of resistance or susceptibility to viral infection. While this approach is often a first step in understanding how a specific sequence is associated with a trait of interest, it does not account for the context of the variants [33]. In actuality SNP are not independent of one another, due to linkage disequilibrium. The association of a haplotype, or functional block of sequence, is the proper approach to determine associations with complex phenotypes such as viral response. The increased significance of the haplotype approach in association studies has been shown with ApoE variants and its association with Alzheimer's disease, in which the haplotype structure analysis identified the causative protein variant [34] and in transmembrane xenobiotic transporters with two or more amino acid variants [35]. These examples are structurally analogous to Mx, where the haplotype structure is vital to the consideration of functional variants' association with viral response [23].

Mx has at least 4 functional domains, each playing a key role in the protein's ability to exert an antiviral function. The GTPase activity in the N-terminal G-domain has long been recognized as required for Mx activity [16]; however, the exact mechanism by which GTPase activity disrupts virus replication is still unknown. In studies by Schusser et al. [14] chicken Mx was cloned and expressed from chicken embryo fibroblast cells from White Leghorn type chickens genotyped as homozygous for the resistance allele (MxCDS1892-A) or the susceptible allele (MxCDS1892-G). In addition to reporting no difference in antiviral activity, they also reported no detectable GTPase activity. In the current study White Leghorn type chickens have at least 4 haplotypes with MxCDS1892-A and 3 with MxCDS1892-G, and at least 5 different SNP combinations within the G-domain, and no alleles that only differ at MxCDS1892. Currently it is unclear how many, if any, of the different chicken Mx haplotypes have GTPase activity, or which variants within the G-domain may affect GTPase activity. Curiously, the SNP identified as under selection and/or associated with performance traits appear to be located around the edge of the GTPase active site.

In addition to the GTPase activity, Mx functions as part of a large complex oligomer dependent on key secondary and tertiary structural elements. These oligomers are made up of 16 Mx dimers that form a large ring around viral ribonucleoprotein complexes wherein the G-domain's enzymatic activity delivers its antiviral effects [23]. Studies by Gao *et al.* [23], [29] have begun to elucidate the key amino acids critical for the formation of this complex quaternary structure for human MxA and have determined that most of these residues are located within the MD and GED regions. The GED region and specifically loop 4 also appear to play a role in defining Mx viral specificity [30]. Across the 9 elite lines examined here, 9 SNP were identified in this region, 4 dNS and 5 dS. The majority of the dS SNP appear to be in close proximity to residues described to be important for oligimerization of human MxA, and even includes a dS SNP (MxCDS1248) that was associated with performance traits. The GED is also where the “resistance allele” is located (MxCDS1892). The overall significance of these SNP variants on Mx function, either individually or within a haplotype is still unclear. Given the numbers of SNP identified across genetic lines, evaluation of chicken Mx functionality will require better consideration of the haplotypes instead of SNP in isolation.

Historical analysis of the haplotype frequencies within the lines evaluated herein indicated that there has been a significant shift in the haplotypes present within six of these lines. Simultaneously, these lines are under intensive selection for numerous traits related to egg production, general animal health, and resistance to MDV. The change in frequency of specific haplotypes is correlated with genetic progress in these lines, suggesting specific advantage of certain haplotypes. The associations found between Mx haplotype and various production traits are interesting. Many common avian viral diseases are known to cause mild to severe reduction in egg production, decrease appetite, depress the immune system and affect the physiology of the reproductive tract. Anti-viral properties of Mx variants could be providing enhanced resistance to viruses routinely encountered throughout the lifecycle of a bird, consequently providing a slight overall improvement in performance.

Review of previous reports of chicken Mx sequence diversity and its functional role in viral resistance provides few conclusive answers. This current work has focused on developing a comprehensive understanding of the significance of sequence diversity of the Mx gene in multiple lines of chicken with multi-generation genotypes and extensive production trait information. Among these lines additional sequence variants were identified that had not been described previously, and more importantly new discrete haplotypes were observed whose frequency appears to be under selection across multiple generations. Thus it is apparent that in future studies of chicken Mx the complete haplotype of the gene should be considered as the functional unit rather than a single SNP.

In addition to the haplotype, it is also important to understand these differences in context of their location within the three dimensional structure of the mature protein. The important functional components of the mature protein can be identified either in structural modeling studies or by evaluating the selection pressures on individual residues as described. These variant sites may provide insight into how Mx functions in the response to virus. The degree of variation contained within the chicken Mx, particularly within commercial stocks, is counter to preconceptions that commercial stocks are highly inbred with limited variability. These levels of diversity within the commercial lines provide vast opportunities for subsequent functional studies. The identification of two novel recombinants within these lines indicates that novel variation does arise and can be maintained within highly selected commercially utilized genetic lines.

Collectively, these data represent the most exhaustive survey of genetic diversity within the Mx gene of commercial layer-type chickens. In addition to the identification of novel SNP, this data reports the association of Mx SNP with both disease resistance and performance traits, and highlights the need for a better understanding of the haplotypes formed by all of the SNP. Mx is a large, complex protein with multiple functional domains. Each domain plays a role in the Mx oligomer and its ultimate function in the host. Understanding how these various SNP and haplotypes interact with each other to function properly in the cell will be key to our understanding the role Mx plays in the interferon-mediated response to viral infections in chickens.
